# Pim-3 as a potential predictor of chemoradiotherapy resistance in locally advanced rectal cancer patients

**DOI:** 10.1038/s41598-017-16153-3

**Published:** 2017-11-22

**Authors:** Rong-xin Zhang, Zhong-guo Zhou, Shi-xun Lu, Zhen-hai Lu, De-sen Wan, Zhi-zhong Pan, Xiao-jun Wu, Gong Chen

**Affiliations:** 1 State Key Laboratory of Oncology in Southern China, Guangzhou, China; 2 Department of Colorectal Surgery, Sun Yat-sen University Cancer Center, Guangzhou, China; 3 Collaborative Innovation Center of Cancer Medicine, Guangzhou, China; 4 Department of Pathology, Sun Yat-sen University Cancer Center, Guangzhou, China; 5 Department of hepatobiliary surgery, Sun Yat-sen University Cancer Center, Guangzhou, China

## Abstract

Approximately 30% of locally advanced rectal cancer patients might not benefit from chemoradiotherapy; however, the response to neoadjuvant chemoradiotherapy in these cases is difficult to predict. Pim-3 is a member of the provirus integration site for a moloney murine leukemia virus family of proteins that contributes to cell proliferation, survival, and chemotherapy resistance. Therefore, the relationship between Pim-3 expression and response to neoadjuvant chemoradiotherapy in rectal cancer patients is important to evaluate. 175 rectal cancer patients who underwent neoadjuvant treatment enrolled in this study. The relationship between Pim-3 expression on immunohistochemical analysis of rectal cancer tissue, which was obtained before treatment, the response to chemoradiotherapy and survival was investigated. The patients with no Pim-3 expression were more likely to achieve a pathologic complete response to chemoradiotherapy than patients with Pim-3 expression (*P* = 0.001). Cox multivariate analysis showed that the significant prognostic factors were Pim-3 expression (*P* = 0.003) and the number of neoadjuvant chemotherapy cycles (*P* = 0.005) for overall survival. Neoadjuvant chemotherapy cycles (*P* = 0.007), adjuvant chemotherapy cycles (*P* = 0.004) and pathology types (*P* = 0.049) were significant prognostic factors for disease-free survival. Pim-3 is a potential predictive biomarker for the response of rectal cancer to chemoradiotherapy.

## Introduction

Colorectal cancer is the fifth most commonly diagnosed cancer and fifth cause of cancer death in China in both men and women^1^. In China, the incidence of rectal cancer is higher than that of colon cancer^2^. In patients with rectal cancer, especially the locally advanced type, the local recurrence rates even after radical resection are high and were reported to range from 15% to 16%^3^ and to be associated with poor prognosis^3,4^. Several randomized controlled clinical trials demonstrated that chemoradiotherapy could reduce the local recurrence rate of rectal cancer, reduce the tumor mass, and increase the tumor resection rate^5,6^. Based on these results, neoadjuvant chemoradiotherapy has been recommended by both the ESMO and NCCN guidelines^6,7^ to improve the prognosis of locally advanced or stages II and III resectable rectal cancer^8^. Over the past decade, the use of neoadjuvant chemoradiotherapy has dramatically increased in China.

The effects of neoadjuvant chemoradiotherapy have usually been determined by histopathologic investigation using tumor regression grading (TRG) systems, such as those by Mandard, Becker, Dworak, and Rodel^9^. The Ryan grading system, which is based on the degree of post-neoadjuvant treatment regressive changes, including fibrosis, and the percentage of residual tumor, was recommended by the NCCN guideline and AJCC. In most cases, a complete response or subtotal tumor regression after neoadjuvant treatment is associated with better patient outcomes^10^. However, the pathologic complete response (pCR) rate following neoadjuvant treatment is reported to be low at 10–20%^11^. Previous studies on locally advanced rectal cancer patients five years after neoadjuvant treatment have reported 65.2–76% overall survival rates, 52.2–68% disease-free survival rates, 6–10.7% local recurrence rates, and 34.4–36% distant metastasis rates^6,12,13^. Therefore, neoadjuvant treatment for rectal cancer remains to be optimized.

Selection of the most suitable patients for chemoradiotherapy is difficult for oncologists. Neoadjuvant treatment may delay the opportunity for radical surgical resection in approximately 30% of rectal cancer patients who would not benefit from chemoradiotherapy and may even lead to progression of the tumor or metastasis during treatment^3^. All selection criteria used by doctors are based on pelvic magnetic resonance imaging (MRI) and ultrasound colonoscopy findings, such as invasion of all layers of the rectal wall and metastases to regional lymph nodes and the mesorectal fascia. Establishing more effective, objective markers that identify patients who are unlikely to benefit from neoadjuvant treatment would have a great impact in clinical practice.

The Pim family of kinases, including Pim-1, Pim-2, and Pim-3, promotes the inactivation of the pro-apoptotic protein Bad by phosphorylation. Pim is an oncogene that has anti-apoptotic functions and collaborates with the proto-oncogene Myc to cause tumor growth. The Pim proteins can regulate tumor proliferation and the cell cycle as well as enhance the anti-apoptotic functions of some normal and tumor cells^14–17^. Previous research has indicated that Pim kinase promotes the transition of the cell cycle from the G1 phase to the S phase and accelerates cell proliferation^14,18,19^. Until now, very few studies on the function of Pim-3 in metastasis or the response to treatment of colorectal cancer can be found^20–22^. Our previous study indicated that Pim-3 is expressed in colorectal cancer tissue at a rate of approximately 32.6%, but it was very rare in normal colorectal tissue (0.02%)^23^. We also demonstrated that patients who were positive for Pim-3 had a poor prognosis and showed minimal response to chemotherapy^23^.

We hypothesized that Pim-3 expression in rectal cancer tissue may be associated with chemotherapy resistance; however, no prior studies have reported on the relationship between response to chemoradiotherapy and Pim-3 expression in rectal cancer. Therefore, the present study aimed to detect Pim-3 expression in rectal cancer and the response to chemoradiotherapy in such cases.

## Results

### General characteristics

**Figure 1:**
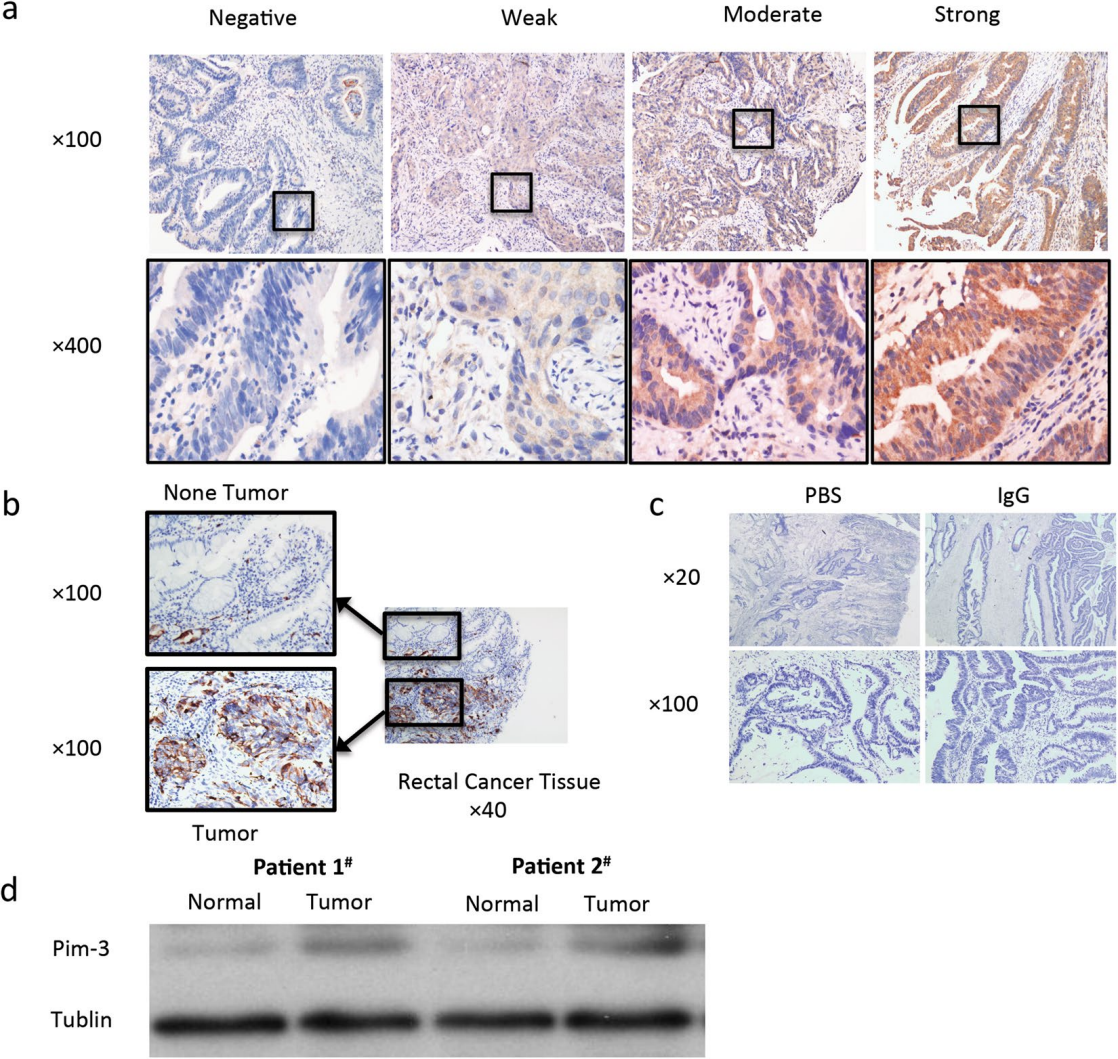
(**a**) Different expression levels of Pim-3 protein in the biopsy tissue before neoadjuvant chemoradiotherapy of 175 rectal cancer patients. The level of Pim-3 expression was classified as follows: negative, weak, moderate, and strong. (Immunohistochemical staining, ×100 and ×400). (**b**) Example of Pim-3 expression in the tumor/non-tumor tissue on the same slide. (Immunohistochemical staining, ×100 and ×200). (**c**) The comparison between using PBS and IgG as negative control. (**d**) Pim-3 expression in rectal cancer and normal tissue by western blot analysis.

This study enrolled 175 patients with pathologically confirmed rectal cancer who received neoadjuvant chemoradiotherapy; of these, 130 patients demonstrated Pim-3 expression in the primary tumor, while 45 patients were negative for Pim-3 expression (Table 1 and Fig. 1a). According to our IHC result, Pim-3 was rarely expressed in the normal tissue (Fig. 1b). No difference was found between the Pim-3-positive and Pim-3-negative groups, except for the distribution of tumor regression grading (TRG) (*P* = 0.01). Of all patients, 85 patients were defined as chemoradiotherapy responders and 90 patients were defined as non-chemoradiotherapy responders. Positive lymph nodes (P = 0.008), perineural invasion (PNI) (P = 0.014) and cycles of neoadjuvant chemotherapy (P = 0.014) were significant different between two groups. Further details are presented in Table 2.

**Table 1:** Clinical and pathologic characteristics of the Pim-3-negative and Pim-3-positive patients.

Characteristic	Pim-3 negative (n = 45)				Pim-3 positive (n = 130)				P value
	No.	%	Mean	SD	No.	%	Mean	SD	
Sex:									0.369
Male	33	73.3			81	62.3			
Female	12	26.7			49	37.7			
Age			53.93	13.38			55.65	11.72	0.415
T stage*:									0.533
T2	0	0			5	3.8			
T3	25	55.6			63	48.5			
T4b	20	44.4			62	47.7			
N stage*:									0.289
N negative	16	35.6			59	45.4			
N positive	29	64.4			71	54.6			
LN number			7.11	4.18			7.75	5.748	0.492
Metastasis LN									0.832
Yes	6	13.3			19	14.6			
None	39	86.7			111	85.4			
PNI									0.791
Yes	1	2.2			6	4.6			
None	44	97.8			124	95.4			
TD									0.115
Yes	0	0			8	6.2			
None	45	100			122	93.8			
LVI									0.791
Yes	1	2.2			6	4.6			
None	44	97.8			124	95.4			
Pathology types									
Highly differentiated ADC	1	2.2			1	0.8			0.469
Middle differentiated ADC	38	84.4			110	84.6			
Poorly differentiated ADC	5	11.2			10	7.7			
Undifferentiated ADC	1	2.2			9	6.9			
TRG									**0.01**
0	23	51.1			22	16.9			
1	10	22.2			30	23.1			
2	12	26.7			77	59.2			
3	0	0			1	0.8			
Survival status:									0.505
Alive	40	88.9			112	86.2			
Dead	5	11.1			18	13.8			
CEA			8.8	18.6			15.4	31.7	0.195
Ca 19-9			22.7	35.9			37.1	94.4	0.320
Neo-chemo regime:									0.533
None	0	0			2	1.5			
Capecitabine	7	15.6			23	17.7			
CAPOX	38	84.4			99	76.2			
FOLFOX	0	0			5	3.8			
5-FU	0	0			1	0.8			
Neo-chemo cycles:									0.368
0	0	0			2	1.5			
1	0	0			1	0.8			
2	20	44.4			67	51.5			
3	7	15.6			27	20.8			
4	18	40			33	25.4			
Adjuvant chemotherapy:									0.433
None	7	15.6			22	16.9			
Capecitabine	2	4.4			18	13.8			
CAPOX	35	77.8			85	65.4			
FOLFOX	1	2.2			4	3.1			
5-FU	0	0			1	0.8			
Adjuvant chemotherapy cycles:									0.558
0	7	15.6			22	16.9			
1	4	8.9			10	7.7			
2	9	20			15	11.5			
3	3	6.7			20	15.4			
4	10	22.2			29	22.3			
5	2	4.4			8	6.2			
6	10	22.2			22	16.9			
8	0	0			4	3.1			
Surgical procedure:									0.327
AR	33	73.3			85	65.4			
APR	12	26.7			40	30.8			
Hartmann	0	0			5	3.8			

*The T and N stages were based on MRI before surgery. TRG = tumor regression grading; 5-FU = 5 fluorouracil; AR = anterior resection; and APR = abdominal perineal resection. Neo-chemo: Neoadjuvant chemotherapy. ADC: Adenocarcinoma. LN: lymph nodes. PNI: Perineural invasion. LVI: Lymphovascular invasion.

**Table 2:** Clinical and pathologic characteristics of the chemoradiotherapy responder and chemoradiotherapy non-responder groups.

Characteristic	Chemoradiotherapy responder group (n = 85)				Chemoradiotherapy non-responder group (n = 90)				P value
	No.	%	Mean	SD	No.	%	Mean	SD	
Sex:									0.404
Male	58	68.2			56	62.2			
Female	27	31.8			34	37.8			
Age			55.47	11.5			54.97	12.7	0.782
T stage*:									0.204
T2	3	3.5			2	2.2			
T3	48	56.5			40	44.4			
T4b	34	40			48	53.3			
N stage*:									0.459
N negative	25	29.4			22	24.4			
N positive	60	70.6			68	75.6			
LN number			6.75	4.6			8.38	5.9	
LN Metastasis									**0.008**
Yes	79	92.9			71	78.9			
None	6	7.1			19	21.1			
PNI									**0.014**
Yes	85	100			83	92.2			
None	0	0			7	7.8			
TD									0.084
Yes	84	98.8			83	92.2			
None	1	1.2			7	7.8			
LVI									0.143
Yes	84	98.8			84	93.3			
None	1	1.2			6	6.7			
Pathology types of ADC									0.773
Highly differentiated	0	0			2	2.2			
Middle differentiated	74	87.1			74	82.2			
Poorly differentiated	7	8.2			8	8.9			
Undifferentiated	4	4.7			6	6.7			
Survival status:									0.331
Alive	76	76.5			76	72.2			
Dead	9	23.5			14	27.8			
CEA			10.8	26.4			16.4	31.1	0.208
Ca 19-9			22.5	33.3			43.7	111.2	0.093
Neoadjuvant chemotherapy regimen									0.508
None	0	0			2	2.2			
Capecitabine	12	14.1			18	20			
CAPOX	72	84.7			65	72.2			
FOLFOX	1	1.2			4	4.4			
5-FU	0	0			1	1.1			
Neoadjuvant chemotherapy cycles:									0.014
0	0	0			2	2.3			
1	0	0			1	1.1			
2	33	38.8			54	60			
3	22	25.9			12	13.3			
4	30	35.3			21	23.3			
Adjuvant chemotherapy regimen									0.387
None	14	16.5			15	16.7			
Capecitabine	8	9.4			12	13.3			
CAPOX	62	72.9			58	64.4			
FOLFOX	1	1.2			4	4.4			
5-FU	0	0			1	1.2			
Adjuvant chemotherapy cycles:									0.310
0	14	16.5			15	16.7			
1	7	8.3			7	7.8			
2	17	20			7	7.8			
3	11	12.9			12	13.3			
4	19	22.3			20	22.2			
5	5	5.9			5	5.6			
6	11	12.9			21	23.3			
8	1	1.2			3	3.3			
Surgical procedure:									0.235
AR	55	64.7			63	70			
APR	29	34.1			23	25.6			
Hartmann	1	1.2			4	4.4			
Pim-3 expression									**0.001**
Negative	33	38.8			12	13.4			
Weak	30	35.3			36	40			
Moderate	15	17.7			29	32.2			
Strong	7	8.2			13	14.4			

*The T and N stages were based on preoperative MRI. TRG, tumor regression grading; 5-FU, 5 fluorouracil; AR, anterior resection; APR, abdominal perineal resection; ADC: adenocarcinoma; LN: lymph node; PNI: perineural invasion; LVI: lymphovascular invasion.

### Response to chemoradiotherapy

In the logistic regression analysis, Pim-3 expression showed a statistically significant relationship with the pCR [risk ratio (RR) = 5.132, 95% confidence interval (CI): 2.442–10.787; *P* = 0.001] and response to chemoradiotherapy (RR = 4.47, 95% CI: 1.94–10.018; *P* = 0.001).

### Overall survival and disease-free survival

All patients were followed-up until August 15^th^, 2017. The 175 patients with 39 months of median follow-up were enrolled in the survival analysis. At the end of follow-up, 148 patients were alive, and 27 had died. Cox multivariate analysis showed that the significant prognostic factors were Pim-3 expression (RR = 1.991, 95% CI = 1.255–3.159; *P* = 0.003) and the number of neoadjuvant chemotherapy cycles (RR = 0.762, 95% CI = 0.630–0.921; *P* = 0.005) for overall survival. The number of neoadjuvant chemotherapy cycles (RR = 0.520, 95% CI = 0.323–0.838; *P* = 0.007), the number of adjuvant chemotherapy cycles (RR = 0.787, 95% CI = 0.667–0.928; *P* = 0.004) and pathology types (RR = 0.244, 95% CI: 0.060–0.993; *P* = 0.049) were significant prognostic factors for disease-free survival. More details are presented in Table 3.

**Table 3:** Univariate and multivariate analyses of prognostic factors for disease-free survival and overall survival in 175 locally advanced rectal cancer patients who underwent chemoradiotherapy as neoadjuvant treatment.

Variable	DFS				OS			
	Univariate		Multivariate		Univariate		Multivariate	
	RR (95% CI)	P	RR (95% CI)	P	RR (95% CI)	P	RR (95% CI)	P
Sex	1.431(0.734–2.786)	0.292			1.234(0.558–2.729)	0.604		
Age	1.007(0.980–1.034)	0.624			1.021(0.988–1.054)	0.214		
T stage	0.737(0.098–5.567)	0.767			0.998(0.748–2.811)	0.461		
N stage	0.901(0.349–2.327)	0.829			0.898(0.293–2.752)	0.850		
LN	1.012(0.956–1.074)	0.686			0.976(0.904–1.054)	0.541		
Positive LN	2.056(0.934–4.523)	0.073			1.678(0.631–4.457)	0.299		
PNI	2.117(0.639–7.014)	0.220			2.706(0.797–9.186)	0.110		
TD	2.367(0.804–6.963)	0.118			2.246(0.635–7.939)	0.209		
LVI	2.185(0.669–7.134)	0.195			1.610(0.379–6.837)	0.519		
Pathology types	0.244(0.060–0.993)	**0.049**	0.244(0.060–0.993)	**0.049**	0.464(0.104–2.081)	0.316		
TRG	1.432(0.086–2.359)	0.350			1.402(0.179–3.381)	0.425		
Pim-3 expression	1.462(1.119–1.910)	**0.005**			**1.557(1.129**–**2.146)**	**0.007**	1.991(1.255–3.159)	**0.003**
CEA	1.007(0.999–1.015)	0.097			1.002(0.990–1.013)	0.750		
CA 19-9	1.001(0.998–1.004)	0.493			1.001(0.998–1.004)	0.412		
Neo-chemo regime	1.072(0.499–2.306)	0.858			1.242(0.537–2.874)	0.613		
Neo-chemo cycles	**0.527(0.337**–**0.823)**	**0.005**	0.520(0.323–0.838)	**0.007**	0.665(0.419–1.054)	0.082	0.762(0.630–0.921)	**0.005**
Adjuvant chemo	0.221(0.478–1.235)	0.926			0.651(0.412–1.029)	0.085		
Adjuvant chemo cycles	**0.841(0.721**–**0.982)**	**0.028**	0.787(0.667–0.928)	**0.004**	0.760(0.630–0.918)	**0.004**		
Surgical procedure	1.055(0.245–4.534)	0.943			0.239 (0.053–1.087)	0.064		

**Figure 2:**
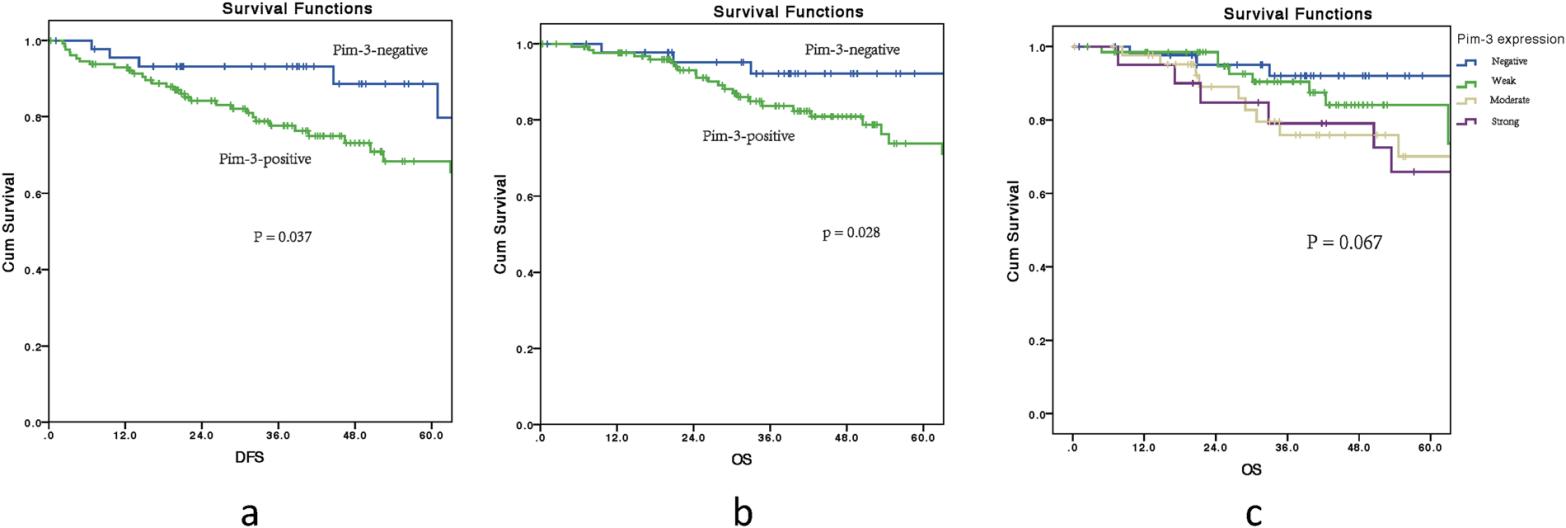
(**a**) Five-year overall survival rate for 137 rectal cancer patients according to Pim-3 expression. (**b**) Five-year disease-free survival rate for 137 rectal cancer patients according to Pim-3 expression. (**c)** 5 years survival curves for 175 locally advanced rectal cancer patients underwent neoadjuvant chemoradiotherapy with different levels of Pim-3 expression. Pim-3 negative was defined as no Pim-3 expression, while weak, moderate, and strong were defined as positive Pim-3 expression. Tumor regression was classified into the following four histologic TRGs based on vital tumor tissue and the ratio of fibrosis after chemoradiotherapy: TRG 0 indicating complete regression and the absence of viable cancer cells; TRG 1 indicating the presence of only small clusters or single cancer cells; TRG 2 indicating the presence of residual cancer cells with predominant fibrosis; and TRG 3 indicating minimal or no decrease in the tumor cells or extensive residual cancer. TRG 0 and 1 patients were considered chemotherapy responders, while TRG 2 and 3 patients were chemotherapy non-responders.

At five years, the overall survival rate for all enrolled patients was 63%, and the disease-free survival rate was 62%. The five-year disease-free survival rate was 73% for the Pim-3-negative and 58% for Pim-3-positive patients (P = 0.037, Fig. 2a) The five-year overall survival rate was 92% for the Pim-3-negative patients and 55% for the Pim-3-positive patients (P = 0.028, Fig. 2b). However, the five-year overall survival rates for different level of Pim-3 expression (negative, weak, moderate, strong) showed no significant (Fig. 2c, P = 0.067).

## Discussion

Neoadjuvant chemoradiotherapy is the standard treatment for patients with locally advanced rectal cancer at stage T3 or T4b with or without regional lymph node metastasis. However, locally advanced rectal cancer can only be diagnosed by MRI and ultrasound colonoscopy. In our previous study, downstaging of the lymph node status (cN+ to post-treatment pN0) was detected in 43 (75%) of 57 patients, and downstaging of the T status was observed in 68 (76%) of 90 patients. In total, 36 (40%) of the patients achieved grade 2 or 3 tumor regression^24^. Based on these results, at least 30% of patients did not benefit from the treatment. Therefore, more objective markers of the response to chemoradiotherapy in rectal cancer patients are needed.

Our previous study showed Pim-3 is expressed differently in rectal cancer tissues and that it could be an important contributor to chemoradiotherapy resistance^23^. Our result confirmed that Pim-3 expression (RR = 4.47, 95% CI: 1.94–10.018; *P* = 0.001) in rectal cancer tissue played key roles in chemoradiotherapy resistance^25^. Locally advanced rectal cancer patients with negative Pim-3 expression were more likely to achieve a better chemoradiotherapy response. Cancer cells have important signal transduction processes that regulate cell death caused by DNA double-strand breaks after ionizing radiation therapy^26^. One of the most likely causes of resistance to chemoradiotherapy is the response to DNA damage; this process is an evolutionarily conserved signaling complex that involves initiation of DNA repair, activation of cell cycle checkpoints, and extensive modulation of gene expression^17,26^. The ataxia-telangiectasia mutated kinase (ATM) is the major protein kinase that plays a key role in the DNA damage response complex via autophosphorylation and recruitment to DNA damage sites to phosphorylate downstream substrates that trigger DNA repair^27^. With irradiation, ATM expression is phosphorylated by Pim-3, which would trigger the activation of DNA damage checkpoints and the phosphorylation of their own substrates to start the DNA damage response.

Furthermore, Pim-3 contributes to chemoradiotherapy resistance by attenuating G2/M cell cycle arrest in cancer cells. ATM can activate the checkpoint kinase (Chk1) and P53 phosphorylation^25^. When exposed to radiation, phosphorylation of Chk1 and phosphatases initiates the G2/M checkpoint to prevent dephosphorylation of CDK1-Cyclin B, which is required for progression into mitosis. The G2/M checkpoint prevents cells with damaged DNA from entering the mitosis phase, wherein the unrepaired DNA double-strand breaks may cause mitotic catastrophe and cell death^28^. When Pim-3 augments the expression of phosphorylated ATM, ATM could allow cells with damaged DNA to enter mitosis and escape cell death. We hypothesized that Pim-3 overexpression induces the activation of ATM, which subsequently activates Chk1, leading to augmentation of DNA repair through cell cycle arrest and the DNA repair pathways. Therefore, a tumor that expresses a high level of Pim-3 may be more resistant to chemoradiotherapy. More research is needed to investigate the complex mechanisms of chemoradiotherapy resistance.

In the present study, Pim-3 expression, along with the number of neoadjuvant chemotherapy cycles, was demonstrated to be a predictor of prognosis in rectal cancer patients after at least 39 months of follow-up. Our previous study showed that different levels of Pim-3 expression in tumor tissue are associated with different prognoses^23^. This result might be explained by the findings that the patients with Pim-3 expression showed more aggressive biological behavior. The association of Pim-3 with poor prognosis and a stronger capacity for invasion and migration might be explained by the ability of Pim-3 to induce the STAT3 signaling pathway and regulate the expression of apoptosis-related genes and VEGF; these changes trigger the proliferation, differentiation, and apoptosis of cancer cells and genes inhibited the migration and proliferation^29^. Furthermore, Pim-3 kinases cause the phosphorylation of the pro-apoptotic molecule Bad, which promotes its inactivation and increases the expression of the anti-apoptotic family member, Bcl-2. Uncontrolled growth of the tumor would be triggered by these changes. Thirdly, previous research on solid tumors and leukemia^14,15,17^ indicated that Pim-3 expression may have important effects on p53 or on other members of the anti-apoptosis Bcl-2 protein family. As the most important apoptosis-inducing gene in the body, p53 critically influences the mitochondrial and death receptor pathways, which are both important for apoptosis. These are the possible reasons for the aggressive biological behaviors and poor prognosis of rectal cancer patients with high Pim-3 expression.

Some limitations of this study should be considered. First, there were a limited number of subjects in this study. A randomized, clinical trial should be conducted to provide additional data that will support our conclusions. Prior to chemoradiotherapy, more tissue samples should be obtained for detecting KRAS and BRAF mutations.

In conclusion, this study showed that Pim-3 expression in rectal cancer was associated with poor response to chemoradiotherapy and poor prognosis. The mechanism underlying these results may be related to the Pim-3 pathway, but this requires further investigation. Pim-3 is a potential predictive maker of the response to chemoradiotherapy in rectal cancer.

## Methods

### Patients

Patients who received neoadjuvant chemoradiotherapy for pathologically confirmed rectal cancer between May 1, 2005 and May 1, 2016 were enrolled in this study. Exclusion criteria were multiple primary colorectal cancer sites, the inability to complete standard neoadjuvant chemoradiotherapy, death within one month after surgery, not receiving radical surgical resection, and incomplete information. Prior to neoadjuvant treatment, tumor tissue obtained by colonoscopy was deposited in the tissue bank of Sun Yat-sen University Cancer Center. Adjuvant chemotherapy was recommended for all patients, including those who achieved pCR.

This study protocol was approved by the Sun Yat-sen University Cancer Center ethics committee and complied with the national legislation and the Declaration of Helsinki. Informed consent was obtained from all participants included in the study.

Of these, 114 were men and 61 were women. Tumor (T) staging and lymph node metastasis were assessed by MRI. The stages were T2 in 5 patients, T3 in 88 patients, and T4b in 82 patients. Lymph nodes were positive for metastasis in 100 patients and negative in 75 patients. All patients received standard radiotherapy, with a total dose of 50 Gy delivered to the gross tumor volume and 46 Gy delivered to the clinical target volume. High energy photons (6 or 8 MV) were used simultaneously in 25 daily fractions, from Monday to Friday, over a period of approximately five weeks. Radiotherapy was combined with chemotherapy in all patients except two.

The neoadjuvant chemotherapy regimen consisted of capecitabine in 30 patients, capecitabine and oxaliplatin (CAPOX) in 137 patients, FOLFOX in 5 patients, and 5-fluorouracil (5-FU) in 1 patient. Chemotherapy was given for one cycle in 1 patient, two cycles in 87 patients, three cycles in 34 patients, and four cycles in 51 patients. Surgical resection involved anterior resection in 118 patients, abdominal perineal resection in 52 patients, and Hartmann resection in 5 patients.

### Tumor regression grading

**Figure 3:**
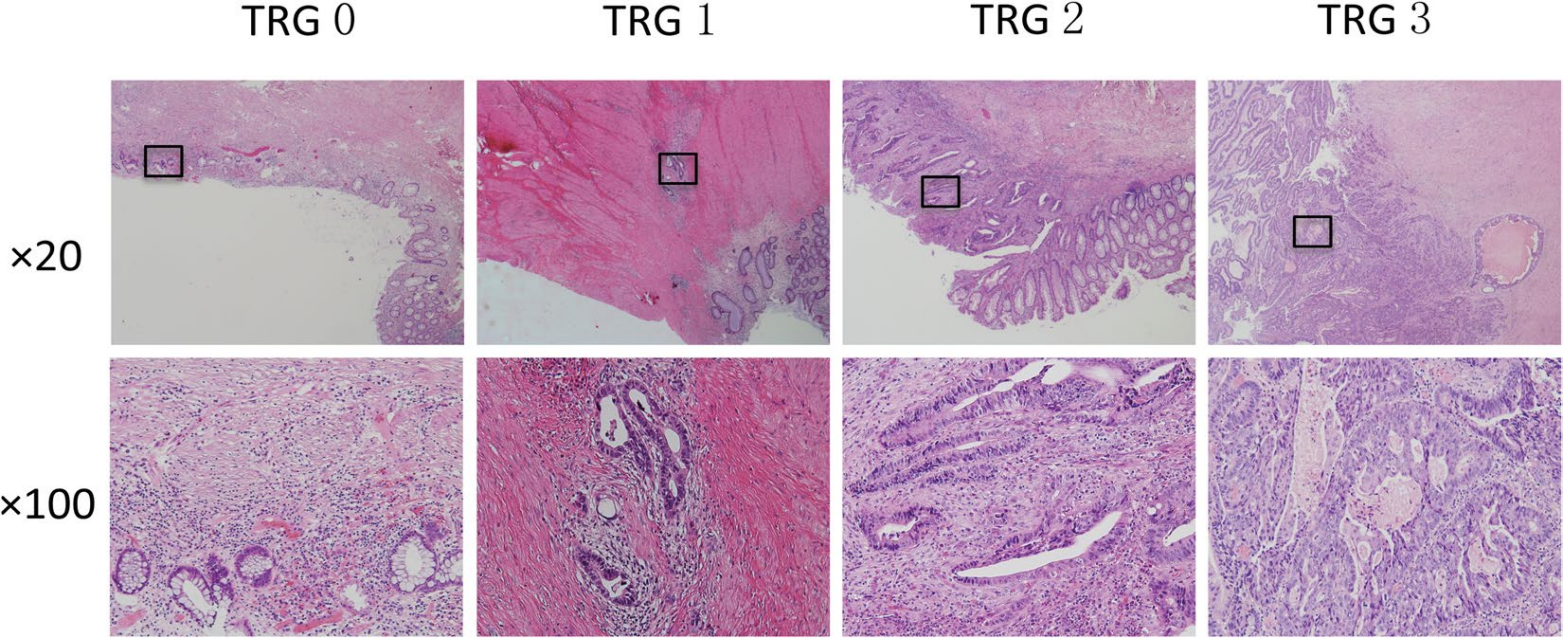
AJCC tumor regression grading (TRG) was classified into four histological TRGs, based on vital tumor tissue at the ratio of fibrosis: TRG 0 as complete regression and the absence of viable cancer cells; TRG 1 as the presence of only small clusters or single cancer cells; TRG 2 as the presence of residual cancer cells, but with predominant fibrosis; and TRG 3 as minimal or no decrease in tumor cells or extensive residual cancer.

The AJCC TRG system was used in this study to determine the effects of chemoradiotherapy. Tumor regression was classified into the following four histologic TRGs based on vital tumor tissue and the ratio of fibrosis after chemoradiotherapy: TRG 0 indicating complete regression and the absence of viable cancer cells; TRG 1 indicating the presence of only small clusters or single cancer cells; TRG 2 indicating the presence of residual cancer cells with predominant fibrosis; and TRG 3 indicating minimal or no decrease in the tumor cells or extensive residual cancer (Fig. 3). TRG 0 and 1 patients were considered chemotherapy responders, while TRG 2 and 3 patients were chemotherapy non-responders.

### Follow-up

All patients were followed up in the outpatient department every three months in the first two years, every six months for the next three years, and annually after five years. During each visit, the patients underwent physical examination; testing for CEA and CA 19-9; and abdominal and pelvic ultrasound examinations. Colonoscopy after resection was recommended at approximately one year or at three months if not performed preoperatively because of an obstructing lesion. A repeat colonoscopy was typically recommended at three years and every five years thereafter, unless follow-up colonoscopy indicated the presence of an advanced adenoma in which case the colonoscopy was repeated in one year. All patients underwent chest, abdominal, and pelvic CT scans annually until five years after surgery.

### Immunohistochemical analysis and scoring

Immunohistochemistry was performed based on our previously reported standard procedure^23^. Paraffin-embedded specimens were serially cut into three sections with a thickness of 4 μm. One section was used for routine hematoxylin and eosin staining, and the other two sections were used for staining using the streptavidin peroxidase (SP) immunohistochemistry method. IgG was used as the negative control (when PBS was used as negative control, we got nearly the same result, Fig. 1c). The experimental procedure was performed according to the instructions of the reagent kit manufacturer. After incubation for 30 minutes at 60 °C, the tissue sections were deparaffinized with xylene, rehydrated, and immersed in distilled water for 5 minutes. After blocking, the sections were incubated with the primary antibody, secondary antibody, and enzyme-labeled SP. The sections were developed using DAB and counterstained with hematoxylin. After staining, the sections were cleared, mounted, and subjected to microscopy. The major reagent was a goat anti-human Pim-3 polyclonal antibody (Santa Cruz, CA, USA) at a concentration of 1:200. A positive Pim-3 signal was localized to the cytoplasm. Five representative high power field images of each section were selected, and the number of positive cells in 500 cells was counted.

Each slide was evaluated by applying the immunohistochemistry (IHC) scoring system that was used in our previous study^30^. Two pathologists reviewed all pathology results. If their conclusions were different, a third pathologist independently evaluated the case and decided on the final result. According to the intensity of positive cells, the results were classified as follows: negative, weak, moderate, and strong^23^. Negative was defined as no Pim-3 expression, while weak, moderate, and strong were defined as positive Pim-3 expression.

### Western blotting analysis

Cells were lysed with RIPA lysis buffer (PBS, 1% NP-40, 0.5% sodium deoxycholate, 0.1% SDS), which containing 10 mg/ml aprotinin, leupeptin, and 1 mM phenylmethylsulfonyl fluoride. Samples were put on ice for 30 min incubation, and then were centrifuged at 18,000 g for 15 min to remove insoluble materials. Protein concentration in supernatant was measured by Bradford Protein assay. A total of 30 μg protein was resolved by 10% SDS polyacrylamide gel, and then transferred to a PVDF membrane. After transfer, PVDF membrane was blocked by 5% nonfat milk in Tris-buffered saline, then incubated with a mouse monoclonal antibodies against Pim-3 (1:200; Santa Cruz Biotechnology, Texas, USA) and a mouse monoclonal antibodies against GAPDH (1:5000; Proteintech, USA) overnight at 4 °C with gentle shaking. Then, proteins were detected with horseradish peroxidase-conjugated secondary antibody for 1 hour at room temperature, followed by the ECL development (Bio-Rad Laboratories, Hercules, CA, USA). The western blot analysis was used to confirm the result of IHC (Fig. 1d).

### Statistical analysis

The clinical and follow-up data were analyzed using SPSS v19.0. The χ^2^ test, continuity correction χ^2^ test, and Fisher’s exact test were used to assess the baseline variables of the patients. The significance of the variables was tested using multivariate Cox’s regression model and logistic regression model. Overall survival was defined as the interval between surgical resection and death or end of follow-up. Disease-free survival was defined as the interval between surgical resection and recurrence, metastasis, or end of follow-up. A *P*-value of <0.05 was considered statistically significant.

### Data availability statement

Supporting data are available to Editorial Board Members and referees.

### Ethical approval and informed consent

This was a retrospective study that had been approved by the Sun Yat-sen University Cancer Center Institutional Review Board on Medical Ethics (ethics committee approval number GZR2015-133). All patients offered written informed consent for possible future data analysis before treatment.

## Electronic supplementary material

Supplementary Information
