# Supplementary material for: Pim-3 as a potential predictor of chemoradiotherapy resistance in locally advanced rectal cancer patients
Source: Sci Rep. 2017 Nov 22;7:16043. doi: 10.1038/s41598-017-16153-3 (PMC5700084; doi:10.1038/s41598-017-16153-3)

Pim-3 as a potential predictor of chemoradiotherapy resistance in locally advanced rectal cancer patients

Rong-xin Zhang[1,2,3], Zhong-guo Zhou[1,3,5], Shi-xun Lu[1,2,4], Zhen-hai Lu[1,2,3], De-sen Wan[1,2,3], Zhi-zhong Pan[1,2,3], Xiao-jun Wu[1,2,3], Gong Chen[1,2,3]

[1]State Key Laboratory of Oncology in Southern China

[2]Department of Colorectal Surgery, Sun Yat-sen University Cancer Center

[3]Collaborative Innovation Center of Cancer Medicine, Guangzhou, China

[4]Department of Pathology, Sun Yat-sen University Cancer Center

[5]Department of hepatobiliary surgery, Sun Yat-sen University Cancer Center

Supplementary materials

Supplemental figure 1. Proliferation of colon cancer cells suppressed by inhibition of pim3 kinase

Assay format: Cell proliferation MTS assay.
Assay Protocol: The CellTiter 96 AQueous One Solution Cell Proliferation Reagent (Promega) was used according to manufacturer protocol. 4000 HCT-116 cells were seeded into each well of the 96-well plate. After twenty-four hours for cells attaching, cells were treated with AZ1208 with or without 5-FU for 3 days. Plates were incubated for 3 days. Following incubation, 20μl of 3-(4,5-dimethylthiazol-2-yl)-5-(3-carboxymethoxyphenyl)-2-(4-sulfophenyl)-2H-tetrazolium, inner salt (MTS) was added to each well. After incubation for 2 to 4 hours, absorbance at 490 nm was recorded by an MRX revolution plate spectrophotometer (Dynex, Technologies, Chantilly, VA). Averages of at least 5 replicates were plotted and 50% inhibitory concentrations (IC50) were estimated based on sigmoidal curve fitting.

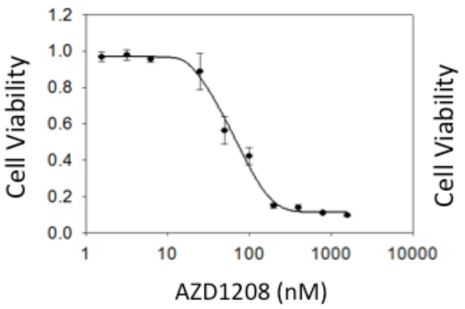

Live cells were Measured by MTS assay. Log(dose)-response curves for human colon cancer HCT116 cell line are shown as labeled. Each data point represents the mean of at least 5 replicates; Error bars represent 95% confidence intervals. Each IC50 was calculated based on sigmoidal curve fitting to the repective data set (IC_{50} = 68 nM).

Supplemental Figure 2. Expression of Pim-3 after silencing by siRNA (Examined by western blot).

Cell culture

The human colorectal cancer cell line HCT116 was obtained from the American Type Culture Collection. The cells were cultured in McCoy-5A(Gibco, Gaithersburg, MD, USA) containing 10% fetal bovine serum(Gibco ,Grand Island, NY, USA) at 37□in 5% of CO2 and saturated humidity. The cells were tested regularly for mycoplasma (New MycoProbe Mycoplasma Detection Kit, R&D Systems). Western Blot was performed to determine the expression of Pim-3 after silencing by siRNAs targeting human PIM-3(Supplementary Table 1) , which were synthesized by GenePharma (Suzhou, Jiangsu, China). For transient transfection, siRNAs were transfected using Lipofectamine 3000 (Invitrogen, Carlsbad, CA, USA) according to the manufacturer's instructions. After 24 h of incubation, cells were harvested by trypsinization for further analysis.

For transient transfections, HCT116 cells were seeded into 6-well plates (at 3×105 cells per well, respectively) at 50% to 60% confluence. After 24 hours, the cells were transfected with oligonucleotides at a concentration of 20 nmol/L using Lipofectamine 3000 (Invitrogen) according to the manufacturer's instructions. After 6 hours of incubation at 37°C, the transfection medium was replaced with 2 mL complete medium containing 10% FBS. The cells were collected for real-time PCR and Western blotting at the indicated times.

RNA extraction, reverse transcription and quantitative real-time PCR.

Total cellular RNAs were extracted using TRIzol reagent (Invitrogen) and

transcribed with M-MLV Reverse Transcriptase (Promega, Madison, WI, USA). The mRNA levels were measured by quantitative real-time PCR (qPCR) using a SYBR Green PCR Kit (Bio-Rad Laboratories, Hercules, CA, USA) with specific primers (Supplementary Table 1). GAPDH was used as an internal control. Threshold cycle (Ct) values were normalized against that of the GAPDH internal control. Relative mRNA levels are presented as the 2 −ΔCt value normalized to the control group.

Identifier Type	Sense sequence(5'-3')	Antisense sequence(5'-3')
NC	UUCUCCGAACGUGUCACGU	ACGUGACACGUUCGGAGAA
Pim-3-siRNA #1	CGTGCTTCTCTACGATATG	
Pim-3-siRNA #2	CAAGGCGGACAAGGAGAGC	

Western blotting analysis

Total cell lysates were prepared by extracting proteins with RIPA lysis buffer (PBS, 1% NP-40, 0.5% sodium deoxycholate, 0.1% SDS) containing 10 mg/ml aprotinin, leupeptin, and 1 mM phenylmethylsulfonyl fluoride. After incubation on ice for 30 min, samples were centrifuged at 18,000 g for 15 min to remove insoluble materials. Protein concentration in supernatant was measured by Bradford Protein assay. Using 10% SDS polyacrylamide gel, 30 µg of total protein was resolved by electrophoresis and was then transferred to a PVDF membrane. The membrane was blocked with 5% nonfat milk in Tris-buffered saline followed by incubation with a mouse monoclonal antibodies against PIM-3 (1:200; Santa Cruz Biotechnology, Texas, USA) and a mouse monoclonal antibodies against GAPDH (1:5000; Proteintech, USA) overnight at 4°C with gentle shaking. Then, blots were incubated with horseradish peroxidase-conjugated secondary antibody for 1 hour at room temperature. Signals were enhanced with the ECL detection system (Bio-Rad Laboratories, Hercules, CA, USA).

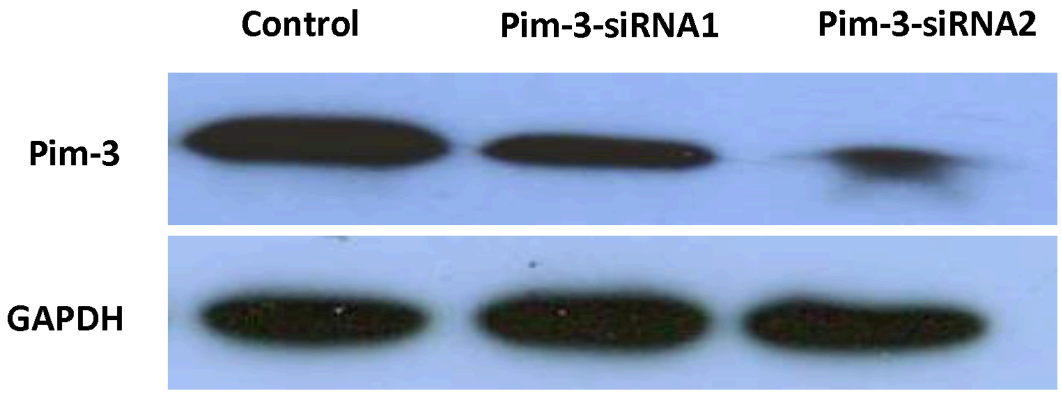

Supplemental Figure 3. Expression of Pim-3 after silencing by siRNA (Examined by Immunohistochemistry).

Immunohistochemistry methods were provided in the main manuscript.

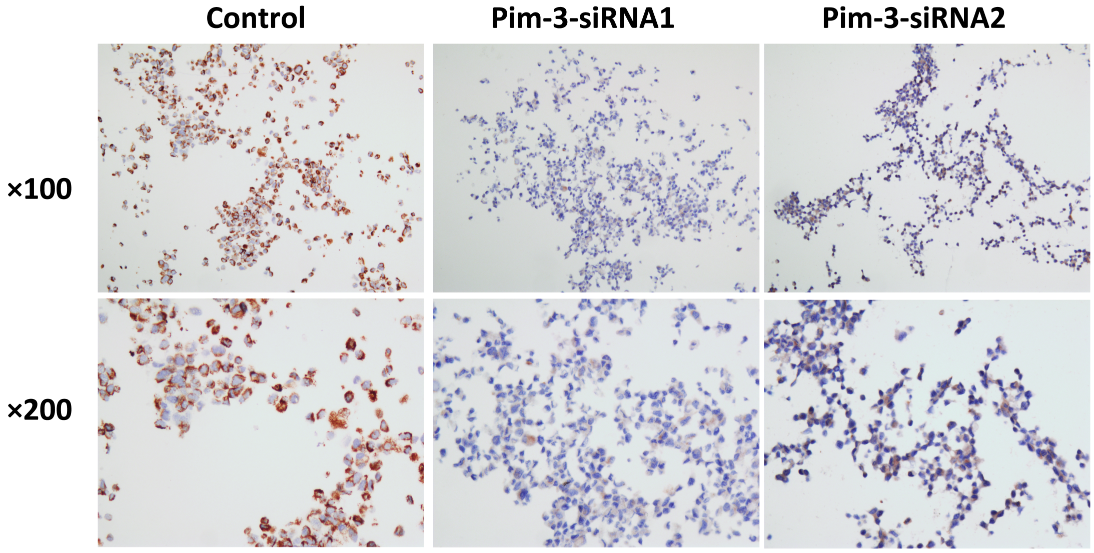

Supplement: Supplementary file 1 — Supplementary Information [file 41598_2017_16153_MOESM1_ESM.pdf]
